# GC/MS and proteomics to unravel the painting history of the lost Giant Buddhas of Bāmiyān (Afghanistan)

**DOI:** 10.1371/journal.pone.0172990

**Published:** 2017-04-05

**Authors:** Anna Lluveras-Tenorio, Roberto Vinciguerra, Eugenio Galano, Catharina Blaensdorf, Erwin Emmerling, Maria Perla Colombini, Leila Birolo, Ilaria Bonaduce

**Affiliations:** 1 Dipartimento di Chimica e Chimica Industriale, Università di Pisa, Pisa, Italy; 2 Dipartimento di Scienze Chimiche, Università degli Studi di Napoli Federico II, Napoli, Italy; 3 Technische Universitaet Muenchen, Lehrstuhl für Restaurierung, Kunsttechnologie und Konservierungswissenschaft, Muenchen, Deutschland; 4 Institute for the Conservation and Promotion of Cultural Heritage, National Research Council of Italy (ICVBC-CNR), Sesto Fiorentino, Italy; 5 Distretto ad Alta Tecnologia dei Beni Culturali (DATABENC Scarl), Napoli, Italy; Bascom Palmer Eye Institute, UNITED STATES

## Abstract

A chemical investigation of the organic paint binders of the Giant Buddhas of Bāmiyān was performed using an analytical approach based on mass spectrometry, combining traditional gas chromatography/mass spectrometry protocols with advanced proteomics methodologies. The research was carried out on a selection of rescued fragments. The data revealed the use of egg proteins as the paint binders of the original layers, in accordance with the traditional use of this proteinaceous medium in antiquity, spanning from the Mediterranean basin to the Far East, and already in the Bronze Age. Egg tempera was thus known to artists of the region in the first centuries AD, probably also due to the position of the Bāmiyān valley, which was connected to the Silk Road. Milk was found in the first historical overpaintings. A new proteomics approach was used, which was able to identify the source of the milk proteins present in the restoration layers, despite their age and degradation. In particular cow’s and goat's milk were both found, in agreement with the documented presence of rich pastures in the Bāmiyān valley when the historical restorations were carried out. Investigating the materials of the Giant Buddhas not only enabled us to obtain isolated data on these invaluable works of art, which are now lost, but contributes to understanding the big “puzzle” of our past and the development of our culture, by implementing and supporting written sources, stylistic and anthropological studies with molecular data.

## Introduction

The Buddha statues of Bāmiyān, which were an outstanding representation of Buddhist Art in central Asian region, became well-known in the West when it was too late to save them. Disregarding international protest, the Taliban destroyed the statues during the war in Afghanistan [1].

**Fig 1:**
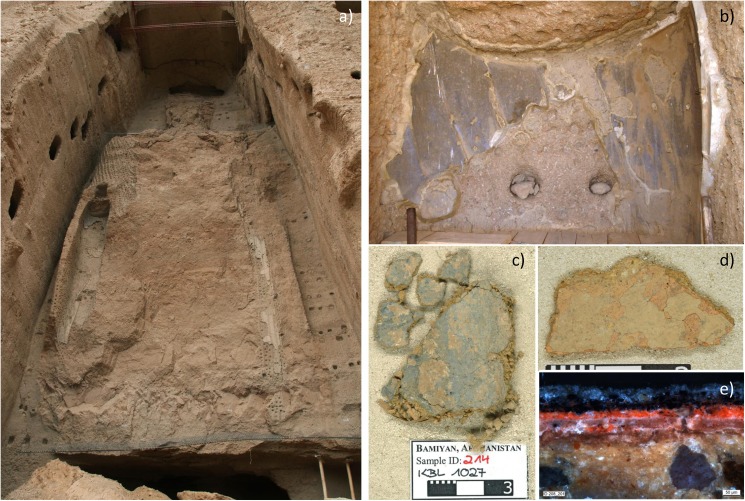
a) The Eastern Giant Buddha after the destruction; b) preserved blue paint layer under the right arm of the Eastern Buddha; c) and d) recovered clay fragments showing painted layers; e) cross-section of one of the samples showing the complex sample build-up consisting in several superimposed colorful paint layers.

After their destruction in March 2001, the activities of the ICOMOS mission started in July 2002 to safeguard the remains and support conservation in the Bāmiyān valley (Fig 1). In 2003, Bāmiyān was included in the World Heritage List and simultaneously placed in the List of World Heritage in Danger. The ICOMOS mission initially focused on rescuing and storing the fragments of the figures, combined with demining the area, and stabilising the niches which were pervaded by cracks due to the force of the explosion [2]. These processes are now almost concluded, but there are still disputes among the international community on how to proceed with the conservation of the Giant Buddhas. Whatever the final decision on how to best tribute the destroyed Buddhas, knowledge of the technology and the materials used in their creation represents a unique opportunity to investigate the original appearance of the statues and their manufacturing techniques.

The giant standing Buddhas of the Bāmiyān valley, which were 35 m and 55 m in height, were the two largest clay statues in the world. On the basis of the AMS 14C dating of the plant material used in their construction, the Eastern and Western Buddhas were built in the period 544 to 595 AD and 591 and 644 AD, respectively [3]. The earliest description of one of the Buddhas dates back to the travelogue *xi you ji* (“The journey to the West”) by the Chinese monk Xuanzang who came to this region in about 630 AD [4]. More accounts of the statues appear in texts by medieval Persian and Arab authors when Bāmiyān was already Islamic and the Buddhist origin of the “idols” had been forgotten. The statues became well-known only in the 19^th^ century when a larger number of Europeans reached Bāmiyān as travellers, adventurers or members of military campaigns. The 20^th^ century brought a scientific approach. Conducted by the Délegation Archéologique Française en Afghanistan (DAFA), examinations were carried out in the 1920s and 1930s. From 1969 to 1978 the Archaeological Survey of India (ASI) in co-operation with the Afghans carried out a comprehensive restoration work on the statues.

The giant Buddhas of Bāmiyān were fundamental pieces of art history not only because of their colossal dimensions, but also because they were precursors and inspirational models that determined the style of clay sculptures in the Far East [5]. Located in a valley that was connected to the Silk Road, and geographically being the westernmost Buddhist site, Bāmiyān was influenced not only by India but also by Sassanian and Byzantine artistic traditions which were transmitted to East Asia [6]. This means that, from a historic-artistic point of view, Bāmiyān is key in studying the west-east influences in ancient Central Asia, as well as the evolution of Buddhist art through the centuries.

Although the most recent photographs of the Giant Buddhas depict them as stone-coloured, the statues once appeared in bright colours surrounded by niches full of colourful, richly detailed murals. In order to apply the pigments–consisting of a fine powder of inorganic or organic coloured material—to the surface of these sculptures, an organic binder must have been used [7]. The organic binder is a film-forming material that disperses the pigment particles, and ensures their adhesion onto the substrate and their cohesion within the paint film. The identification of organic paint constituents in micro-samples from works of art is very challenging from an analytical point of view [8,9], as they are aged and present in relatively low amounts. Several organic and inorganic substances are also often simultaneously present in a layered structure, and can give rise to strong interactions with each other [10–14].

In this paper we present a characterisation of the organic paint binders, which was achieved using an analytical approach based on the combined use of two mass spectrometric techniques: gas chromatography mass spectrometry (GC-MS) and liquid chromatography tandem mass spectrometry LC-MS/MS (Proteomics). The results provided invaluable information on the materials and techniques used, thus helping to preserve the memory of the statues, and improving our understanding of historical livestock, the interchange of cultures and technical know-how of central Asia between the 6^th^ and 8^th^ centuries AD.

## Materials and methods

### Reagents

For the chromatographic technique all the solvents used were Baker HPLC grade. Hexadecane, tridecanoic acid and norleucine, used as internal standards, hexamethyldisilazane (HMDS), and *N*,*O*-bis(trimethylsilyl)trifluoroacetamide (BSTFA) containing 1% trimethylchlorosilane were purchased from Sigma (Milan, Italy). *N*-*tert*-Butyldimethylsilyl-*N*-methyltrifluoroacetamide (MTBSTFA) with 1% trimethylchlorosilane was from Fluka (USA). All reagents and chemicals were used without any further purification. Standard solutions of amino acids in hydrochloric acid (0.1 M), containing 12.5 μmol/mL of proline and hydroxyproline, 1.25 μmol/mL of cysteine and 2.5 μmol/mL of aspartic acid, glutamic acid, alanine, arginine, phenylalanine, glycine, hydroxylysine, isoleucine, histidine, leucine, lysine, methionine, serine, tyrosine, threonine, and valine was purchased from Sigma-Aldrich (USA). A solution containing lauric acid, suberic acid, azelaic acid, myristic acid, sebacic acid, palmitic acid, oleic acid, stearic acid (all purchased from Sigma-Aldrich, USA) in the range of 2–3 μ/g was prepared in isooctane and stored at 4°C.

A polyester resin polymerised by a peroxy organic hardener (Cronolite E.I, Plastiform, Spain) was used for the cross-section preparation. The epoxy resin used for the SR FTIR slices was purchased at Plastiform, Spain.

### Samples

Between 2004 and 2008 under the supervision of the Technical University of Munich, Chair of Conservation, Art Technology and Conservation Science (TUM rkk), conservators recovered with the help of locals approximately 10000 fragments from the rubble at the feet of the statues.

The work was coordinated by ICOMOS Germany, under the direction of UNESCO, and was conducted in co-operation with Afghan authorities, as the governor of Bamiyan Province, the Director of Historical Monuments, and the Ministry of Culture. The salvaging of the fragments was part of the work to clear the niches and the space around the feet of the Buddha statues from debris to start the consoldation of the niches' walls. All fragments (including clay, ropes and wooden pegs) were catalogued and stored in shelters and some were selected for scientific investigation. The selection included mostly fragments that were discovered detached from others so the region of the erstwhile statues from where these were derived could not be reconstructed. Few fragments were taken from a context of larger fragments in order to help link the stratigraphy of collected samples to a known part of the statue. Reports on the work on-site can be found in "The Giant Buddhas of Bamiyan. Safeguarding the remains" [15]. Of these, a selection of these samples were exported to Germany under the permission of the Director of Historical Monuments. At present the preservation of the still standing cultural heritage and monuments as well as the protection of the already excavated or salvaged objects and fragments represents a priority. Reconstruction or anastylosis (reassembling of existing but dismembered parts) of at least one of the statues is being decided upon, and when this will be clear, the fragments kept in Germany will return to Bāmiyān, either to be used for reconstruction or to be permanently stored locally.

**Fig 2:**
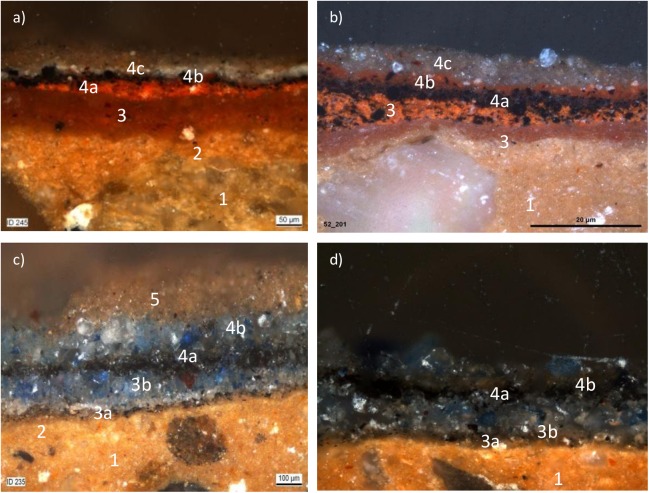
Optical microscope images of the cross-section of samples coming from the garment of the Buddhas. a) and b) red fragments from the *sangati* of the Eastern and Western Buddha, respectively; c) and d) blue fragments from the lining of the Eastern and Western Buddha, respectively.

The paint layers have been investigated in 275 small pieces of painted clay of unknown origin, mostly between 1 and 4 cm in length [16], which were too small to be used for reconstruction purposes. Fragments showed the remnants of several superimposed thick and slightly powdery paint layers, which means that the statues had been repainted over the centuries (Fig 1). The examination of the fragments and cross-sections highlighted that both statues showed the same sample build-up (Fig 2). Underneath the pigmented paint layers, often a preparation/isolation layer (layer 2 in Fig 2) was observed, which seemed to have penetrated the clay surface (layer 1 in Fig 2). In some samples a white non-coherent white priming layer was found. As far as the pigmented paint layers are concerned, it was possible to ascribe them to three to four different historical moments: the lowest was the oldest and probably original layer (layer 3 in Fig 2), and the following two to three layers belonged to overpaintings (layer 4 in Fig 2) performed in subsequent eras (between c. 650 ad 977 AD). On top of the paint layers, almost all samples had a light ochre layer which was attributed to the Indo-Afghan restoration (layer 5 Fig 2).

To summarise what is known about the history of the statues from the scientific investigations that have been performed so far [16], researchers believe that the first preserved and maybe original paint layer of the *sangati—*outer robe—on both statues was pink (white earth + iron oxides) with a dark blue lining (ultramarine). The first historical overpainting resulted in an orange-red (red lead) layer on the previously pink *sangati* of the Western Buddha and a pink overpainting on the Eastern Buddha, which later were both repainted in orange-red (Fig 2). After this overpainting, the *sangati* of both statues was bright orange, while the lining was still blue. The third historical overpainting was carried out on both statues, applying a thin lead white layer which was probably meant to cover the meanwhile severely blackened red lead. The *sangati* of the Western Buddha was then repainted in red (red iron oxide), while on the Eastern Buddha the white underpainting remained visible. The lining was repainted with a paler variety of ultramarine. This is consistent with accounts of authors such as Yakut al Hamawi who in 1218 –just before Genghis Khan destroyed Bāmiyān in 1221 –named them the “red idol” (Western Buddha) and the “white idol” (Eastern Buddha). Both the original paint and the subsequent overpaintings were applied in several layers. No other coloured restoration layers appeared visible in the fragments. This can be explained because in 977 AD Bāmiyān became irrevocably Islamic, and it can be assumed that no further repainting of the statues was carried out after that date. In the 20^th^ century conservation, the statues were covered with a clay suspension, either because of the assumption that the statues were originally “stone-coloured” or to homogenise the patchy appearance caused by partial losses of the polychromy.

In this study, organic paint materials were investigated in 19 of these fragments which were selected as being representative of the different types of paint layer structures discernible on the fragments found and stored in Bamiyan. In an effort to locate the materials in the sample build-up, several sub-samples (a total of 45), containing one or more paint layers, were obtained by manually scratching each fragment with a scalpel. Each subsample (on average in the range of 0.1–1.5 mg) was then analysed separately in order to determine the chemical composition of each layer or group of layers. A detailed description of the samples analysed is reported in S1 and S2 Files [16].

### GC/MS analysis

The analytical procedure adopted is based on a wet chemical pre-treatment of the sample, which includes extractions, desalting and hydrolysis steps, in order to separate three fractions from the same micro-sample to be analysed separately by GC-MS: a saccharide fraction (i.e. a solution of per-silylated di-thioacetal derivatives of aldoses and uronic acids, derived from the hydrolysis of saccharide materials), an amino acidic fraction (i.e. a solution of tert-butyldimethylsilyl derivatives of amino acids, derived from the hydrolysis of proteins), and a lipid resinous fraction (i.e. a solution of trimetylsilyl derivatives of acids, alcohols as well as neutral compounds, derived from the hydrolysis of lipids, resins and waxes). The method is described in detail in the literature [17]

A 6890N GC system gas chromatograph coupled with a 5973 mass selective detector single quadrupole mass spectrometer equipped with a split-splitless and a 6890N GC system gas chromatograph (Agilent Technologies, Palo Alto, CA) coupled with a 5975 mass selective detector (single quadrupole mass spectrometer equipped with PTV injector, by Agilent Technologies) were used. The MS transfer line temperature was 280°C; the MS ion source temperature was kept at 230°C; and the MS quadrupole temperature was at 150°C. For the gas chromatographic separation, an HP-5MS fused silica capillary column (5% diphenyl/95% dimethyl-polysiloxane, 30 m X 0.25 μm i.d., 0.25 μm film thickness, Agilent Technologies) with a deactivated silica precolumn (2 m X 0.32 μm i.d., J) was used. The carrier gas was used in the constant flow mode (He, purity 99.995%) at 1.2 mL/min. The mass spectrometer operated in the EI positive mode (70 eV) and MS spectra were recorded both in TIC (total ion current) and SIM (single ion monitoring) mode.

The PTV injector and the chromatographic oven were programmed as follows:

Lipids-Resins. The PTV injector was used in splitless mode at 280°C and the oven was: 80°C isothermal for 2 min, 10°C/min up to 200°C, 200°C isothermal for 3 min, 10°C/min up to 280°C, 280°C isothermal for 3 min, 20°C/min up to 300°C isothermal for 30 min.

Amino acids. The PTV injector was used in splitless mode at 220°C, and the oven was: initial temperature 100°C isothermal for 2 min, then 4°C/min up to 280°C, 280°C isothermal for 15 min.

Saccharides. The PTV injector was used in splitless mode at 250°C and the oven was: 50°C isothermal for 2 min, 5°C/min up to 190°C at, 190°C isothermal for 20 min 5°C/min up to 280°C, 280°C isothermal for 15 min. -for saccharides: 50°C isothermal for 2 min, 5°C/min up to 190°C at,190°C isothermal for 20 min 5°C/min up to 280°C, 280°C isothermal for 15 min.

#### Proteomic analyses

Proteinaceous material was identified following a minimally invasive proteomic analytical procedure as described in detail the literature [18], except for a preliminary incubation of the sample in strongly protein denaturing conditions (6M Urea) which were introduced in order to favour the exposure of the proteinaceous material to the action of proteases, as also reported in a previous work [19].

Briefly, microsamples (ca 100–500 μg) were incubated for 1 h in 20 μL in 6 M urea followed by sonication for 30 min at room temperature. The samples were then 6-fold diluted with ammonium bicarbonate 10 mM pH 7.5 and enzymatic digestion carried out by the addition of 1 μg of trypsin at 37°C for 16 hours. The supernatants were then recovered by centrifugation, filtered on 0.22 μm PVDF membrane (Millipore), concentrated and purified using a reverse-phase C18 Zip Tip pipette tip (Millipore). Peptides were eluted with 20 μL of a solution made up of 50% Acetonitrile, 0.1% Formic acid in Milli-Q water and analysed by LC-MS/MS. The eluate was analyzed by LC-MSMS on a 6520 Accurate-Mass Q-Tof LC/MS System (Agilent Technologies, Palo Alto, CA, USA) equipped with a 1200 HPLC System and a chip cube (Agilent Technologies). After loading, the peptide mixture was first concentrated and washed on a 40 nl enrichment column (Agilent Technologies chip), with 0.1% formic acid in 2% acetonitrile as the eluent. The sample was then fractionated on a C18 reverse-phase capillary column (Agilent Technologies chip) at a flow rate of 400nL/min, with a linear gradient of eluent B (0.1% formic acid in 95% acenonitrile) in A (0.1% formic acid in 2% acetonitrile) from 3% to 80% in 50 min.

Peptide analysis was performed using data-dependent acquisition of one MS scan (mass range from 300 to 2000 m/z) followed by MS/MS scans of the three most abundant ions in each MS scan. MS/MS spectra were measured automatically when the MS signal exceeds the threshold of 50,000 counts. Each LC-MSMS analysis was preceded and followed by blank runs to avoid carry-over contamination.

Double and triple charged ions were preferentially isolated and fragmented. The acquired MS/MS spectra were transformed into *mzData* (.XML) format and used for protein identification with a licensed version of MASCOT (www.matrixscience.com) version 2.4. with 10 ppm MS tolerance and 0.6 Da MS/MS tolerance; peptide charge from +2 to +3. No fixed chemical modification was inserted, but possible oxidation of methionines, formation of pyroglutammic acid from glutamine residues at the N-terminal position of peptides, and deamidation at asparagines and glutamines were considered as variable modifications (Leo et al., 2011) in order to query SwissProt and NCBI databases, without any taxonomy restriction. Only proteins presenting two or more peptides were considered as positively identified.

Selected Ion Monitoring (SIM) analysis was used to improve the quality of the fragmentation of selected ions.

Protein sequences were aligned using Align tool available online at UniProt (24), which uses Clustal-Omega program (25).

## Results

Although no treatises on painting sculptures are known from Afghanistan, the position of the Bāmiyān valley in connection to the Silk Road suggested possible organic binders such as those indicated in Indian texts and found in clay sculptures and mural paintings from different parts of Asia [20–26] and the Mediterranean basin [27–30]. The paintings of the adjacent caves in Bāmiyān were also investigated [31,32]. Several organic materials were thus possible, including polysaccharide, glycerolipid and proteinaceous materials as adhesives, binders and consolidants, while resins and waxes were possibly used as materials in subsequent restorations.

On these basis, a GC/MS procedure was used in an initial phase, in order to identify polysaccharide, proteinaceous, glycerolipid materials, as well as waxes and terpenoid resins in the same micro-sample. The procedure is based on a multistep chemical pre-treatment of the sample, in order to obtain three different fractions to be analysed separately by GC-MS: an amino acidic, a saccharide, and a lipid-resinous fraction. The procedure allows to identify proteinaceous, polysaccharide, and glycerolipid materials by means of the quantitative analysis of amino acids, aldoses and uronic acids, mono- and dicarboxylic aliphatic acids, respectively. Waxes and terpenoid resins are identified by the recognition of specific molecular patterns [17].

Drying oils, waxes and terpenoid resins were not detected in any of the samples analysed. Saccharide materials—tragacanth gum and honey—were seldom identified in some of the subsamples (S3 File). Saccharide material was found in some of the samples, and the sugar profiles obtained from each sample analysed are presented in S3 File. Tragacanth gum was identified on the basis of the presence of fucose in the samples [29].

**Fig 3:**
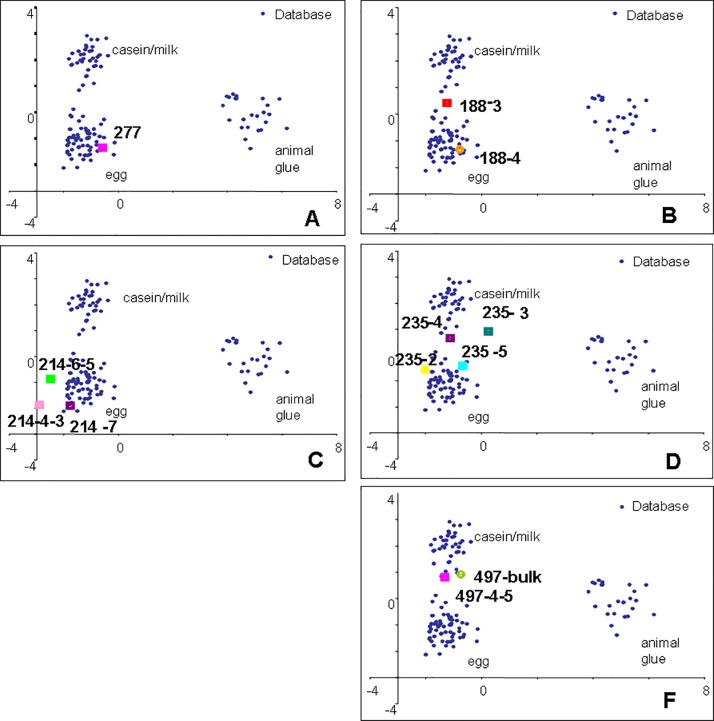
PCA score plots of samples from the Eastern Buddha. A) 277; B) 188; C) 214; D) 235; F) 497

**Fig 4:**
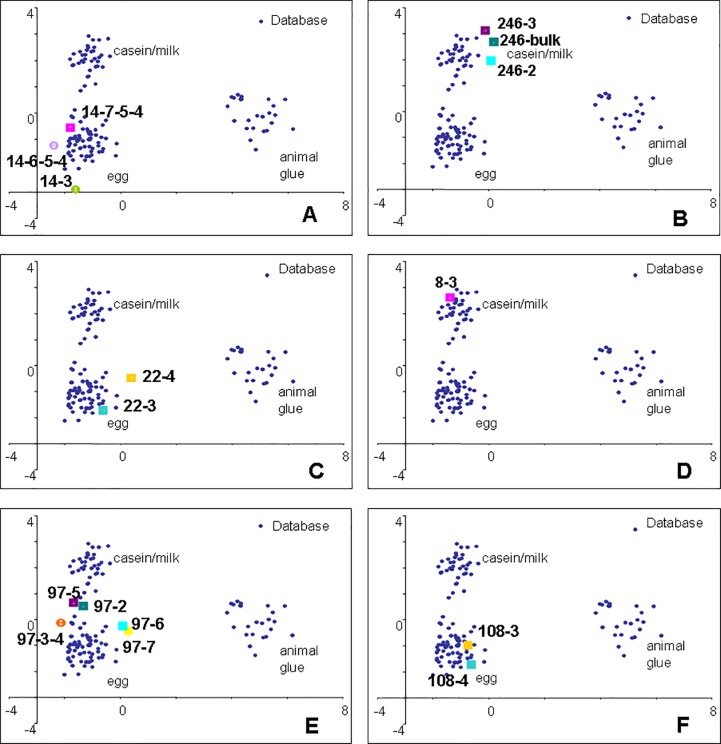
PCA score plots of samples from the Western Buddha. A) 14; B) 246; C) 22; D) 8; E) 97; F) 108.

**Fig 5:**
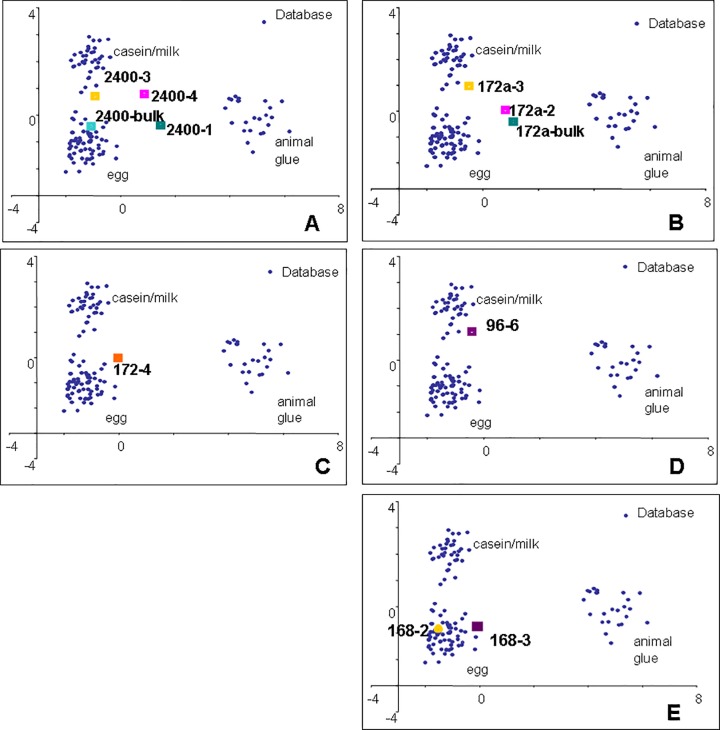
PCA score plots of samples from the Western Buddha. A) 2400; B) 172a; C) 172; D) 97; E) 168.

In all fragments proteinaceous matter was present. The recognition of proteinaceous paint binders by GC/MS is based on the detection of amino acids obtained by microwave-assisted hydrolysis of the proteins extracted from the samples, and purified from pigments by solid phase micro-extraction on C4 beds [33]. With the exception of animal glue, which contains hydroxyproline, the proteins present in organic paint media all contain the same set of amino acids. Identification is thus based on the statistical comparison of the quantified amino acidic profile of the sample with a database of profiles of reference samples [33]. In particular the quantitative percentage content of amino acids of each sub-samples determined was subjected to a multivariate statistical analysis together to a data-set of 121 reference samples of animal glue, egg and casein, using the principal components analysis (PCA) method. The graphical representations of the statistical treatment of the data used to assist the protein identification (PCA score plots) are shown in Figs 3–5. The amino acidic profiles obtained from each sample analysed are presented in S4 File.

The data clearly identified egg and milk in some of the sub-samples, although the amino acidic profiles of some of the samples remained unidentified.

To better characterise the proteinaceous binders, a selection of 30 sub-samples (obtained from 11 different fragments were analysed by proteomics procedures (S1 and S2 Files).

Although proteomics analytical methods are well established in the life science field, their application in the field of cultural heritage is relatively new, with the first paper dating back to the early 2000s [34] and specific methodological adaptations are still in progress. The analytical procedure used in this study proved capable of detecting and identifying proteins in cultural heritage samples, in the presence of pigments and even when degraded [18,35]. The samples were subject to enzymatic digestion with trypsin, and the obtained mixtures of peptides were purified and concentrated using reverse-phase C18 pipette tips and analysed by LC-MS/MS [4]. Proteins were confidently identified in 16 out of the 30 sub-samples analysed (Table 1, and details of the identifications are given in S5 File).

**Table 1:** Milk proteins identified in the samples from Buddhas of Bāmiyān by LC-MSMS. Proteins were identified by searching UniprotSprot database with MSMS Ion search Mascot software (Matrix Science) with Chordata as the taxonomy restriction, with deamidation on Gln and Asn, oxidation on Met, pyro-Glu formation at Gln at the N-terminus of peptides as variable modifications. Only proteins identified with at least two peptides were considered as significant. Details of the identifications are reported in S5 File.

protein	sample															
	214–2	206-4-3	206-7-4	188–4	235-bulk	235–5	235–3	235–4	235–1	214–7	214–4	18–4	18–1	22–4	16–4	16–3
**Alpha-S1-casein**	X	X		X	X	X	X	X	X	X	X	X	X	X	X	X
**Alpha-S2-casein**	X		X		X		X			X	X					
**Beta-casein**	X				X		X			X	X	X				X
**Beta-lactoglobulin**	X				X					X	X					

The proteomics approach led to the identification of proteins based on the recognition of specific *m/z* values and fragmentation patterns of enzymatically digested peptides.

The presence of several milk proteins, i.e. Alpha S1 casein, Alpha S2 casein, Beta casein and Beta lactoglobulin, enabled us to confidently assess that milk had been used extensively. Alpha S1 casein was identified in almost all the samples where a positive identification was observed, namely in 15 out of the 16 positive samples, while Alpha S2 casein was identified in 6 samples, Beta casein in 7, and Beta lactoglobulin in 4 samples (Table 1).

Very interestingly, proteomics analyses enabled us to establish the coexistence of milk from cows and from goats. We identified four possible sources for the milk (*Bos taurus*, *Bubalis bubalis*, *Capra hircus* and *Ovis aries*) (S5 File), which resulted in multiple possible assignments with very similar scores, given the high percentage of similarity between the homologous proteins from different sources. Table 2 reports the list of the peptides from milk proteins that were identified in the whole set of samples. Some peptides are in common between proteins from different organisms, while some are unique for a single sequence, thus allowing species identification. We will now focus on the peptides of alpha S1 and alpha S2 caseins (Table 2), since these are the proteins that were identified in the largest number of cases with a high confidence level.

**Table 2:** Identified peptides of alpha S1-casein and alpha S2-casein. A full list of matched peptides that were observed in the whole set of samples. The position of the starting and ending residues of each peptide are indicated as superscripts. The sequences that are unique to the species are shown in bold; the accession numbers in the UniProt database are shown in brackets.

Protein	*Bos taurus*(P02662)	*Capra hircus*(P18626)
**Alpha-S1-casein**	^99^EDVPSER^105^	^99^EDVPSER^105^
	^106^YLGYLEQLLR^115^	^106^YLGYLEQLLR^115^
	^107^LGYLEQLLR^115^	^107^LGYLEQLLR^115^
	^19^**HQGLPQEVLNENLLR**^37^	^38^**FVVAPFPEVFR**^**4**8^
	^29^**EVLNENLLR**^37^	
	^38^**FFVAPFPEVFGK**^49^	
	^50^**EKVNELSK**^57^	
	^121^**VPQLEIVPNSAEER**^134^	
**Alpha-S2-casein**	***Bos taurus* (P02663)**	***Capra hircus* (P33049)**
	^96^ALNEINQFYQK^106^	^96^ALNEINQFYQK^106^
	^141^EQLSTSEENSK^151^	^141^EQLSTSEENSK^151^
	^130^**NAVPITPTLNR**^140^	^62^**NANEEEYSIR**^72^
	^153^**TVDMESTEVFTK**^164^	^130^**NAGPFTPTVNR**^140^
	^189^**FALPQYLK**^196^	^213^**TNAIPYVR**^220^
	^215^**VIPYVRYL**^222^	

**Fig 6:**
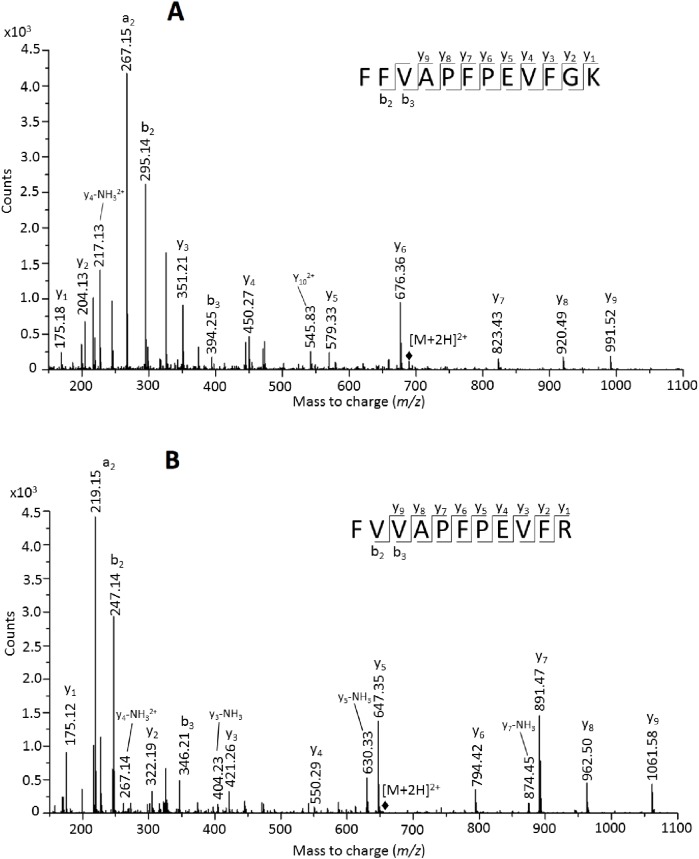
MSMS spectra, as acquired in SIM analysis mode, of the doubly charged ions at m/z 692.87. (A) of the peptide ^38^FFVAPFPEVFGK^49^ of alpha S1 casein from *Bos taurus* (P02662) and 654.58 (B) of the peptide ^38^FVVAPFPEVFR^48^ of alpha S1 casein from *Capra hircus* (P18626), respectively, which were identified in the LC-MS/MS analysis of the 214-clay sample

The peptide FFVAPFPEVFGK of alpha S1 casein was detected in 15 samples and is a unique peptide for bovine alpha S1 casein (either from *Bos taurus* (P02662) or from *Bubalus bubalis* (O62823)). Similarly, the peptide FVVAPFPEVFR is unique of caprine alpha S1 casein (either from *Capra hircus* (P18626) or from *Ovis aries* (P04653)). The concomitant identification of peptides that are unique to either *Bos taurus* and *Capra hircus* homologous proteins suggested that both cattle and goat's milk were used. In the alignment of the sequences of the four alpha S1 caseins considered above (see S5 File Fig 6), these two sequences were aligned, and their co-presence in the sample indicates the coexistence of the homologous proteins in mixture.

As confirmation of the effective presence of the two unique peptides in the same peptide mixture, we selectively searched for them by running selected samples in SIM mode, and manually interpreting the MS/MS spectra. For example, Fig 6 reports the fragmentation spectra of the ions that were interpreted as peptide ^38^FFVAPFPEVFGK^49^ of alpha S1 casein from *Bos taurus* (P02662, UniProt entry) and peptide ^38^FVVAPFPEVFR^48^ of alpha S1 casein from *Capra hircus* (P18626), respectively, as detected in the LC-MS/MS analysis in one of the samples. Both spectra can be manually inspected and unambiguously interpreted, thus confirming the assignments to the peptides inferred above.

A similar reasoning was made for the peptide ^130^NAVPITPTLNR^140^ from alpha S2 casein from *Bos taurus* (P02663) and peptide ^130^NAGPFTPTVNR^140^ from alpha S2 casein from *Capra hircus* (P33049) which are unique for the two protein sequences, respectively, and are aligned in the multiple alignment of the identified alpha S2 caseins (S5 File). Thus the co-presence of milk from cows and goats was confirmed as expected on the basis of the peptides of alpha S1 casein reported above.

With respect to GC/MS based approaches, proteomics univocally identify the protein. On the other hand, chemical modifications occurring to a variable extent in paint layers upon ageing [11–14], can still impair positive protein identification [36]. In other words, if the detection of specific peptides univocally identifies a protein in a sample, the non-identification of specific peptides does not necessarily rule out the presence of a protein. The egg binder, identified by GC/MS but not by the proteomics approach, could possibly be degraded to such an extent as to prevent the detection of any of the peptides, as expected on the basis of the protein sequences. It has also been demonstrated that oligosaccharide moieties of albumen and egg yolk proteins seem to create a molecular hindrance which, by preventing the accessibility of proteases, greatly impairs the identification process of egg [37].

## Discussion

The attribution of the organic materials to the different paint layers was possible thanks to the analysis of a high number of samples and their division into sub-samples. Synchrotron light FTIR imaging on paint cross sections was also attempted. However, given the relatively high content of pigments and the degraded state of the organic binders, it mainly provided information on the distribution of inorganic components. By combining the results obtained by GC/MS and proteomics, and considering the sample build-up, it is possible to draw some general conclusions.

Egg and a saccharide material identified as tragacanth gum were found in the clay modelling (sample 277, GC/MS), while milk was found in the plaster layers on top (samples 18-1and 97–2, GC/MS; samples 18-1235-1 and 214–2, proteomics).

The isolation/preparation layer shows the presence of egg (samples 14–3, 108–3 and 168–3, GC/MS). It was not possible to determine the binder of the inhomogeneous priming layer. Despite the difficulties in isolating under the microscope the original paint layers, avoiding interferences from upper layers, it was possible to identify egg both the pink layer (sample 97-3-4, GC/MS) and the dark blue one (sample 235–2, GC/MS). The first historical restoration was done using milk as binder, both for the *sangati* (samples 246–3, 8–3, 97–5, GC/MS; samples 18–4, 22–3, 16–4, 16–3, 206-4-3, proteomics) and the lining (samples 235–4, 235–2, 497-4-5, GC/MS; 235–5, 235–4, 235–3, 214-3-4, proteomics). The following historical restorations are more difficult to interpret, given their patchy distribution and the resulting inhomogeneity of the sample stratigraphies. Egg (samples 22–4, 97–6, 97–7, 108–4, 172–4, GC/MS), milk (sample 2400–3, 172a-3, 96–6, GC/MS; 22–4 proteomics), and tragacanth gum (samples 188–4, 235–6, 14-7-4-5, 108–4, 108–3, 172–4, 96–6) were found. Samples from the Indian restoration were also analysed (samples 235–6, 214–7, 497–7, 2400–4, 172a-4, GC/MS; 18–8, 16–7, and 214–7, proteomics). Proteomics techniques did not identify any proteinaceous material. Traces of proteins were found in two of the samples by GC-MS techniques (214–7 and 2400–4), but a contamination from the layers below cannot be excluded, as the same composition of the layer underneath was always found. Tragacanth gum was found in two of the samples (235–6 and 214–7). These results seem to indicate that the Indian restoration was done with a saccharide binder.

The data provide important technical information on Asian art of the time. Although the stylistic relations between the Hellenistic and Roman world (and their successors) in the West, and the Sogdian and Persian art, as well as Indian and even Chinese in the East, have been discussed and are still studied, the technical aspects are mainly unknown. Egg produces bright paint layers, which are insoluble and elastic, and resist the mechanical stress due to daily and seasonal thermo-hygrometric variations. This was well known in antiquity, both in the East–see for example the polychromy of the Terracotta Army, Xi’an, China (3^rd^ century BC [20])—and in the West–see for example the murals of the Palace of Nestor, Pylos Greece (13^th^ century BC)[27]. Considering the dimensions of the Buddhas, the results indicate that knowledge of the quality of the egg paint and its durability was available to the artists of Buddhist art in central Asia at the time.

Considering the weathering of the most exposed parts of the statues, but also the documented man-made damage such as soot, scratching, and attacks and looting by passing armies (beginning with Genghis Khan), the original paint layers in some areas were probably not well kept after some years. As the statues were in-use religious items held in high esteem, they were well-cared for and kept in good shape, which included overpainting when needed.

The use of milk as paint binder in open-air paintings seems to be a debatable choice instead due to the brittleness of the film formed and its consequent sensitivity to the harsh climatic conditions of the area [38]. The change in the materials used leaves room for debate regarding several important historic-artistic issues. The poorer quality materials could be related to a change in livestock in the area, or to trading along the Silk Road, or to the loss of importance of Bāmiyān as a Buddhist pilgrimage site, as suggested by the lack of any description of the statues by travellers in the area since Xuanzang’s description (7^th^ century) to the 11^th^ century. However Xuanzang’s report of the site in 630 AD mentions that the region produced wheat, fruits and flowers and provided rich pasture for sheep, cattle and horses [39]. The choice of milk might have been based on practical considerations such as the availability of the material in the area.

Proteomics helped us understand the origin of the milk found in the fragments, although it was quite a demanding task, given the age and the degradation undergone by the proteins. In addition, the high degree of similarity between the homologous proteins can lead to multiple possible hits with very similar scores, as highlighted in Table 1 for the case herein reported, where we identified four possible sources of the milk (*Bos taurus*, *Bubalis bubalis*, *Capra hircus* and *Ovis aries*). Only peptides that are unique for *Bos taurus* and *Capra hircus* sequences were detected and since no peptides that were unique to protein sequences from *Bubalis bubalis* and *Ovis aries* were identified, we ruled out the possibility of assessing them as the source of milk. Goat hairs identified in both the undercoat and the finish coat, to strengthen the cohesiveness of the clay layers, further support the presence of goat livestock in the area [40]. Data also clearly showed that one type of milk could not be associated with a specific restoration layer, and in general both types could have been present in the same sub-samples. One possible explanation could be that one type of milk was used for the first restoration and another type for the second, and the binder of the second intervention had penetrated the layers underneath. Another possible and more likely explanation is that, considering the gigantic dimensions of the surfaces being painted, and the multi-layered structure of the paints, restorers used whatever they had, mixing milk from different animals, or using any milk they had available when they were painting the different layers.

Drying oils were not identified in any of the samples collected from the statues, although they were clearly identified in the paintings in the adjacent caves [31]: this might be related to the dimensions of the statues, or because of stylistic choices, or because different artists were travelling in Central Asia using different painting techniques.

Interesting is also the identification of tragacanth, which is a natural gum obtained from the dried sap of several species of Middle Eastern legumes of the genus Astragalus, including A. adscendens, A. gummifer, A. brachycalyx and A. tragacanthus [41]. The presence of the Astragalus species in Afghanistan since the first realization of the statues has already been proven by the identification of Astragalus cuneifolius Bunge as the material used to manufacture the ropes used as a core of the fold ridges from the Western Buddha [42].

## Conclusions

In this study we investigated into the chemical composition of the organic binders used in the lost Giant Buddhas of Bāmiyān, in an effort to learn about these fundamental artefacts of our history, despite the destruction of the war.

The organic materials used as binders and their sources were identified in some of the remaining fragments of the Giant Buddhas. Putting together these data with results of previously performed investigations [15] contributed at improving our understanding of the appearance of the Buddhas over the centuries as well as the materials used. It also enabled us to obtain unprecedented information from a historic-artistic point of view on the livestock, agriculture, trade routes and artistic influences.

Data suggest that the two statues were painted using egg in the original paint layers, confirming its use in polychrome artworks all around the Indo-European continent in ancient times. However, the data also highlighted a change in the materials used in the overpaintings, as milk and some pigments of a poorer quality were used in the first historical restoration. Proteomics data allowed establishing cows and goats as the source of the milk used as binder.

## Supporting information

S1 File Fragments analysed from the Eastern Buddha. (DOCX)

S2 File Fragments analysed from the Western Buddha. (DOCX)

S3 File GC/MS Saccharide fraction. (DOCX)

S4 File GC/MS Amino acidic fraction. (DOCX)

S5 File LC-MS/MS analyses. (DOCX)
