# Supplementary material for: GC/MS and proteomics to unravel the painting history of the lost Giant Buddhas of Bāmiyān (Afghanistan)
Source: PLoS One. 2017 Apr 5;12(4):e0172990. doi: 10.1371/journal.pone.0172990 (PMC5381772; doi:10.1371/journal.pone.0172990)
Supplement: S1 File — (DOCX) [file pone.0172990.s001.docx]

**GC/MS and Proteomics to unravel the painting history of the lost Giant Buddhas of Bāmiyān (Afghanistan)**

Anna Lluveras-Tenorio, Roberto Vinciguerra, Eugenio Galano, Catharina Blaensdorf, Erwin Emmerling, Maria Perla Colombini, Leila Birolo, Ilaria Bonaduce

**S1 Fragments analysed from the Eastern Buddha**

A total of 6 samples from the Eastern Buddha were analysed by means of GC/MS and Proteomics. The samples were taken from fragments mainly attributed to the folds of the garments as follows: the blue fragments (samples 235,214, 497) can be assigned to the lining of the sangati and the undergarment (uttarasanga); sample 206, that appears reddish, was interpreted as part of the outside of the sangati. Sample 188, however, could not be assigned to any part of the statues.

The sample build-up are described in Table A. The layers analysed as sub-samples for GC/MS and Proteomics are also specified. A bulk sample containing all layers was also analysed by GC/MS (sample 497) and by Proteomics (sample 235).

Table S1. Description of the samples and sub-samples analysed from the Eastern Buddha.

| **Sample ID*** | **Layer** | **(build-up) Description** | **Sub-sample**  **analysed** | **Sub-samples description** | **GC/MS (mg)** | **Proteomics** |
| --- | --- | --- | --- | --- | --- | --- |
| 277 |  | arriccio  (preparation layer underneath the plaster) | 277 | fragment | 6.0 | n.a. |
| 206 | 7 | yellowish claywash  (Indian restoration) | 206-7-6 | scraped material containing layer 7 and layer 6, some contamination from layer 5 | n.a. | xx |
|  | 6 | white layer | 206-7-4 | scraped material containing layer 7 to 4 |  | xx |
|  | 5 | blackened orange |  |  |  | n.a. |
|  | 4 | orange | 206-4-3 | scraped material containing layer 4 and layer 3 |  | xx |
|  | 3 | pink |  |  |  | n.a. |
|  | 2 | white (in traces) |  |  |  | n.a. |
|  | 1 | plaster | 206-1 | scraped material from the surface of the plaster, could contain the traces of white |  | xx |
| 188 | 5 | white layer (few residues) |  |  | n.a. | n.a. |
|  | 4 | yellow ochre layer | 188-4 | scraped material containing some more of layer 4 than3 | 1.2 | xx |
|  | 3 | white gypsum layer | 188-3 | sample flakes, containing more of layer 3 than 4 | 1.7 | n.a. |
|  | 2 | plaster |  |  | n.a. | n.a. |
|  | 1 | arriccio |  |  | n.a. | n.a. |
| 235 | 6 | yellowish claywash  (Indian restoration) | 235-6 |  | 1.0 | n.a. |
|  | 5 | blue paint layer | 235-5 | scraped material | 1.2 | XX |
|  | 4 | dark grey to black underpatinitng | 235-4 | scraped material | 0.3 | xx |
|  | 3 | blue paint layer | 235-3 | scraped material | 0.4 | XX |
|  | 2 | black underpainting | 235-2 | scraped material | <0.1 | n.a. |
|  | 1 | plaster and insulation | 235-1 | scraped material | <0.1 | XX |
|  | 0 | arriccio | 235-0 |  | n.a. | XX |
| 214 | 7 | yellowish claywash  (Indian restoration) | 214-7 | scraped material containing some of layer 6 | 2.5 | xx |
|  | 6 | blue paint layer | 214-5-6 | scraped material containing layer 6 and layer 5 | 1.6 | n.a. |
|  | 5 | black underpainting |  |  |  | n.a. |
|  | 4 | blue paint layer | 214-3-4 | scraped material containing the blue layer and the under painting | 1.3 | XX |
|  | 3 | black underpainting |  |  | n.a. | n.a. |
|  | 2 | plaster | 214-2 | might contain some contamination from layer 3 | n.a. | xx |
|  | 1 | arriccio |  |  | n.a. | n.a. |
| 497 | 7 | Indian clay wash | 497-7 | scraped material from the surface of the fragment | 1.5 | n.a. |
|  | 6 | greyish layer |  |  | n.a. |  |
|  | 5 | blue paint layer | 497-5 | scraped material, some contamination from layer 4 | 0.6 |  |
|  | 4 | black underpainting |  |  | n.a. |  |
|  | 3 | isolation layer |  |  | n.a |  |
|  | 2 | plaster |  |  | n.a. |  |
|  | 1 | arriccio |  |  | n.a. |  |

*These fragments are currently stored at the TUM rkk, but will return to Bamiyan when examinations are finished and a proper storage place at the site will be built. Fragments are publicly available on request, as well as the data obtained from the examinations concluded
