# Supplementary material for: GC/MS and proteomics to unravel the painting history of the lost Giant Buddhas of Bāmiyān (Afghanistan)
Source: PLoS One. 2017 Apr 5;12(4):e0172990. doi: 10.1371/journal.pone.0172990 (PMC5381772; doi:10.1371/journal.pone.0172990)
Supplement: S2 File — (DOCX) [file pone.0172990.s002.docx]

**GC/MS and Proteomics to unravel the painting history of the lost Giant Buddhas of Bāmiyān (Afghanistan)**

Anna Lluveras-Tenorio, Roberto Vinciguerra, Eugenio Galano, Catharina Blaensdorf, Erwin Emmerling, Maria Perla Colombini, Leila Birolo, Ilaria Bonaduce

**S2 Fragments analysed from the Western Buddha**

A total of 13 samples from the Western Buddha were analysed by means of GC/MS, proteomics or both. The samples were taken from fragments mainly attributed to the folds of the garments . Samples that appear reddish (samples 8, 14,16, 18, 22, 97, 168, 246), resulting from layers of pink, orange and red were interpreted as part of the outside of the sangati. Brown samples come from fragments with white surfaces and could not be assigned to any part of the statues tough the stratigraphy of some fragments seems to point of the application of the brown layer on parts of the sangati which had been red before ( samples 108, 96, 172, 172a, 2400).

The sample build-up are described in Table B. The layers analysed as sub-samples for GC/MS and Proteomics are also specified. In some cases, some fragments were analysed as bulk (containing all layers) by GC/MS (samples 246, 2400, 172a), and by proteomics (sample 246 bulk).

Table S2. Description of the samples and sub-samples analysed from the Western Buddha.

| **Sample ID*** | **Layer** | **(build-up) Description** | **Sub-sample**  **(analysed)** | **Sub-samples description** | **GC/MS (mg)** | **Proteomics** |
| --- | --- | --- | --- | --- | --- | --- |
| 18 | 8 | yellowish claywash  (Indian restoration) | 18-8-5 | scraped material. contains layer 8 to layer 5 | n.a. | ++ |
|  | 7 | red layer (incoherent) |  |  |  |  |
|  | 6 | white layer |  |  |  |  |
|  | 5 | blackened orange layer |  |  |  |  |
|  | 4 | orange layer | 18-4 | scraped material. contains layer 4 and a bit of layer 5 |  | ++ |
|  | 3 | pink layer (traces) |  |  |  |  |
|  | 2 | white layer (partially) |  |  |  |  |
|  | 1 | plaster | 18-1 | scraped material from the surface. Contamination from layers 3 and 2 |  | ++ |
| 14 | 8 | Indian restoration (only one point) | - |  | n.a. | n.a. |
|  | 7 | grey layer | 14-7-5-4 | scraped material containing also layers 5 and 4 | 3.3 |  |
|  | 6 | red layer (only one point) | 14-6-5-4 | scraped material | 1.8 |  |
|  | 5 | orange layer |  |  |  |  |
|  | 4 | white layer |  |  |  |  |
|  | 3 | transparent layer | 14-3 | scraped material | 0.9 |  |
|  | 2 | plaster | - |  | n.a. |  |
|  | 1 | arriccio |  |  | n.a. |  |
| 246 | 4 | yellowish claywash  (Indian restoration) |  |  | n.a. |  |
|  | 3 | red/orange layer | 246-3 | red layer, scraped by scalpel | 5.4 | n.a. |
|  | 2 | pink layer (partially) | 246-2 | pink layer, scraped by scalpel | 3.8 |  |
|  | 1 | plaster |  |  | n.a. |  |
| 22 | 6 | Indian restoration |  |  | n.a. | n.a. |
|  | 5 | red paint layer | 22-5 | scraped, contamination from layer 6 | <0.1 | n.a. |
|  | 4 | white layer (very thin) | 22-4 | scraped, containing layer 5 to 3 | <0.1 | ++ |
|  | 3 | orange with discolored surface (black) | 22-3 | scraped material, pure | <0.1 | ++ |
|  | 2 | pink layer | 22-2 | scraped, contamination from layer 1 | <0.1 | ++ |
|  | 1 | isolation layer and plaster | 22-1 | scraped, surface of the clay | 0.1 | n.a. |
| 16 | 7 | yellowish claywash  (Indian restoration) | 16-7-5 | scraped, containing layer 6 and 5 | n.a. | ++ |
|  | 6 | red layer |  |  |  | n.a. |
|  | 5 | white/grey thin layer |  |  |  | n.a. |
|  | 4 | blackened orange layer | 16-4 | scraped |  | ++ |
|  | 3 | orange layer, partly blackened | 16-3 | scraped |  | ++ |
|  | 2 | pink layer |  |  |  | n.a. |
|  | 1 | plaster | 16-1 | scraped from the surface of the clay |  | ++ |
| 8 | 7 | yellowish clay wash  (Indian restoration) |  |  | n.a. | n.a. |
|  | 6 | red layer |  |  | n.a. |  |
|  | 5 | thin white layer |  |  | n.a. |  |
|  | 4 | orange with blackened surface |  |  | n.a. |  |
|  | 3 | orange layer | 8-3 | scraped material | 1.8 |  |
|  | 2 | pink layer |  |  | n.a. |  |
|  | 1 | plaster |  |  | n.a. |  |
| 97 | 7 | red hard layer | 97-7 | scraped material. contains a bit of layer 6 as well | 0.7 | n.a. |
|  | 6 | glue/grey hard layer | 97-6 | scraped material. might contain a bit of layer 7 | 1.7 |  |
|  | 5 | black powdery layer | 97-5 | scraped material. contains a bit of layer 6 | 4.5 |  |
|  | 4 | orange powdery layer | 97-3-4 | scraped material | 0.8 |  |
|  | 3 | pink powdery layer |  |  |  |  |
|  | 2 | plaster | 97-2 | scraped material. might contain some arriccio | 1.2 |  |
|  | 1 | arriccio |  | n.a.(^[[1]](#footnote-1)^) |  |  |
| 108 | 5 | few residues of the Indian restoration |  |  | n.a. | n.a. |
|  | 4 | glossy brown layer | 108-4 | might contain some of the Indian restoration | 2.7 | ++ |
|  | 3 | glossy brown layer | 108-3 | might contain some plaster | 1.3 | n.a. |
|  | 2 | plaster | 108-2 |  | n.a. | ++ |
|  | 1 | arriccio |  |  | n.a. | n.a. |
| 2400 | 4 | clay wash  (Indian restoration) | 2400-4 |  | 1.2 | n.a. |
|  | 3 | brown layer | 2400-3 | the two brown layers are contained | 1.3 | ++ |
|  | 2 | brown layer |  |  | n.a. | n.a. |
|  | 1 | plaster | 2400-1 |  | 1.6 | n.a. |
| 172a | 4 | ochre clay wash  (Indian restoration) | 172a-4 |  | 0.3 | n.a. |
|  | 3 | brown glossy layer | 172a-3 |  | 0.1 | ++ |
|  | 2 | red layer | 172a-2 | containing some plaster from underneath | 0.4 | ++ |
|  | 1 | plaster |  |  | n.a. | n.a. |
| 172 | 4 | brownish layer, semi-transparent | 172-4 | scraped material | 3.8 | n.a. |
|  | 3 | thin red layer (few residues) | - |  | n.a. |  |
|  | 2 | plaster |  |  | n.a. |  |
|  | 1 | arriccio |  |  | n.a. |  |
| 96 | 4 | ochre clay wash | 96-6 | scraped, contamination from the brown layer | <0.1 | n.a. |
|  | 3 | brown glossy layer |  |  | n.a. |  |
|  | 2 | red layer |  |  | n.a. |  |
|  | 1 | plaster |  |  | n.a. |  |
| 168 | 4 | residues of pigmented layer | - |  | n.a. | n.a. |
|  | 3 | transparent preparation layer | 168-3 | scraped material | 1.6 |  |
|  | 2 | plaster | 168-2 | scraped material | 1.3 |  |
|  | 1 | arriccio | - |  | n.a. |  |

*These fragments are currently stored at the TUM rkk, but will return to Bamiyan when examinations are finished and a proper storage place at the site will be built. Fragments are publicly available on request, as well as the data obtained from the examinations concluded

1. n.a. not analysed [↑](#footnote-ref-1)
