# Supplementary material for: GC/MS and proteomics to unravel the painting history of the lost Giant Buddhas of Bāmiyān (Afghanistan)
Source: PLoS One. 2017 Apr 5;12(4):e0172990. doi: 10.1371/journal.pone.0172990 (PMC5381772; doi:10.1371/journal.pone.0172990)
Supplement: S3 File — (DOCX) [file pone.0172990.s003.docx]

**GC/MS and Proteomics to unravel the painting history of the lost Giant Buddhas of Bāmiyān (Afghanistan)**

Anna Lluveras-Tenorio, Roberto Vinciguerra, Eugenio Galano, Catharina Blaensdorf, Erwin Emmerling, Maria Perla Colombini, Leila Birolo, Ilaria Bonaduce

**S3 GC/MS Saccharide fraction**

The glycoside profiles of the sub-samples analysed showing a saccharide content above the detection limit are presented in Table S3. For the sub-samples with a saccharide content between the detection and quantitation limits the presence/ absence (y/-) of the sugars is indicated. The saccharide fraction of some of the fragments was not analysed (497, 246, 2400, 172a).

**Table S3. Glycoside profile of analysed samples and amount of saccharide material.**

| Sample | Sugars | | | | | | | | | Saccharide content  /µg |  |
| --- | --- | --- | --- | --- | --- | --- | --- | --- | --- | --- | --- |
|  | Xyl | Arab | Ramn | Fuc | Gal Ac. | Gluc. Ac. | Gluc | Mann | Galact |  |  |
| 277 | 44.5 | 22.9 | 2.3 | 1.0 | 0.0 | 1.4 | 17.6 | 3.1 | 7.0 | 129 |  |
| 188-4 | 7.8 | 13.6 | 0.5 | 0.8 | 0.6 | 2.1 | 50.8 | 4.0 | 19.8 | 3.56 |  |
| 188-3 | y | y |  |  |  |  | y |  | y | - |  |
| 235-6 | y | y | y | y | - | - | y | y | y | - |  |
| 235-1 | y | y | y | y | - | - | y | y | y | - |  |
| 214 -7 | 10.9 | 3.3 | 1.3 | 1.0 | 0.0 | 0.6 | 46.4 | 13.2 | 23.3 | 2.80 |  |
| 214-6-5 | 7.3 | 4.9 | 1.9 | 1.4 | 0.7 | 1.3 | 34.2 | 15.5 | 32.8 | 5.10 |  |
| 214-4-3 | 47.2 | 12.1 | 2.0 | 1.1 | 0.0 | 0.0 | 0.0 | 0.0 | 37.5 | 0.61 |  |
| 14-7-5-4 | y | y | y | y | - | y | y | y | y | - |  |
| 22-4 | y | y | y | y | - | - | y | y | y | - |  |
| 8-3 | y | y | - | - | - | - | - | - | y | - |  |
| 97-2 | y | y | - | - | - | - | - | - | y | - |  |
| 108-4 | 8.3 | 13.5 | 1.6 | 2.0 | 1.1 | 3.3 | 25.8 | 10.5 | 34.1 | 7.95 |  |
| 108-3 | 11.7 | 12.8 | 0.8 | 1.8 | 0.5 | 0.9 | 31.1 | 7.1 | 33.3 | 1.75 |  |
| 172-4 | 44.1 | 8.8 | 2.3 | 1.7 | 6.7 | 2.2 | 0.0 | 20.0 | 14.3 | 1.79 |  |
| 96-6 | 14.9 | 4.5 | 5.1 | 4.2 | 0.0 | 0.6 | 56.6 | 6.4 | 7.7 | 1.3 |  |
| 168-2 | y | y | - | - | - | - | - | - | y | - |  |
|  |  |  |  |  |  |  |  |  |  |  |  |
