# Supplementary material for: GC/MS and proteomics to unravel the painting history of the lost Giant Buddhas of Bāmiyān (Afghanistan)
Source: PLoS One. 2017 Apr 5;12(4):e0172990. doi: 10.1371/journal.pone.0172990 (PMC5381772; doi:10.1371/journal.pone.0172990)
Supplement: S4 File — (DOCX) [file pone.0172990.s004.docx]

**GC/MS and Proteomics to unravel the painting history of the lost Giant Buddhas of Bāmiyān (Afghanistan)**

Anna Lluveras-Tenorio, Roberto Vinciguerra, Eugenio Galano, Catharina Blaensdorf, Erwin Emmerling, Maria Perla Colombini, Leila Birolo, Ilaria Bonaduce

**S4 GC/MS amino acidic fraction**

In Table S4 the relative amino acid percentage of the samples with an amino acid content above the detection limit is reported together with the amount of proteinaceous material found. Samples 235-6, 235-1, 497-1, 22-5,22-2, 22-1, 172a-4 showed an amminoacidic content below the detection limit and were considered blanks of the procedure.

**Table S4. Ammino acid relative percentage content of the sub-samples analysed and amount of proteinaceous material found**

| **Sample** | **Ala** | **Gly** | **Val** | **Leu** | **Ile** | **Ser** | **Pro** | **Phe** | **Asp** | **Glu** | **Hyp** | **protein content /μg** |
| --- | --- | --- | --- | --- | --- | --- | --- | --- | --- | --- | --- | --- |
| **277** | 8.6 | 13.4 | 8.9 | 12.0 | 7.0 | 7.3 | 6.5 | 6.9 | 16.6 | 12.8 | 0.0 | 2.0 |
| **188-4** | 6.7 | 7.7 | 8.4 | 12.0 | 5.3 | 19.7 | 3.3 | 5.4 | 11.5 | 19.9 | 0.0 | 3.1 |
| **188-3** | 9.6 | 12.4 | 13.3 | 20.8 | 11.5 | 1.4 | 11.8 | 5.6 | 4.7 | 8.9 | 0.0 | 0.3 |
| **235-5** | 9.0 | 3.3 | 7.4 | 12.0 | 6.8 | 7.7 | 5.9 | 13.6 | 10.9 | 23.4 | 0.0 | 1.5 |
| **235-4** | 11.3 | 3.8 | 12.3 | 15.1 | 8.7 | 4.7 | 13.5 | 8.2 | 6.4 | 16.0 | 0.0 | 3.2 |
| **235-3** | 13.8 | 6.9 | 10.8 | 15.6 | 8.8 | 4.4 | 18.9 | 6.3 | 4.8 | 9.8 | 0.0 | 1.0 |
| **235-2** | 13.8 | 6.0 | 10.3 | 15.0 | 9.1 | 6.9 | 10.6 | 7.7 | 5.8 | 14.9 | 0.0 | 2.9 |
| **214-7** | 7.3 | 8.5 | 10.9 | 12.9 | 8.2 | 11.2 | 4.1 | 6.5 | 18.4 | 12.2 | 0.0 | 2.49 |
| **214-5-6** | 5.5 | 6.4 | 9.6 | 11.4 | 7.2 | 11.6 | 5.0 | 5.7 | 17.6 | 20.0 | 0.0 | 3.49 |
| **214-3-4** | 7.4 | 6.9 | 14.4 | 19.0 | 10.7 | 12.2 | 3.6 | 7.1 | 11.7 | 7.1 | 0.0 | 3.85 |
| **497-2** | 5.4 | 8.0 | 12.2 | 14.9 | 8.0 | 5.9 | 7.7 | 4.8 | 10.8 | 22.4 | 0.0 | 1.23 |
| **497-3-2-1** | 5.5 | 7.2 | 10.5 | 11.9 | 6.5 | 5.3 | 9.7 | 5.8 | 12.1 | 25.5 | 0.0 | 0.75 |
| **14-7-6** | 7.4 | 10.3 | 12.5 | 19.1 | 10.3 | 5.9 | 5.3 | 7.5 | 13.6 | 8.1 | 0.0 | 0.78 |
| **14-5-4** | 8.8 | 10.8 | 15.0 | 22.9 | 11.3 | 4.4 | 5.5 | 7.1 | 7.6 | 6.7 | 0.0 | 0.54 |
| **14-3** | 16.0 | 15.5 | 18.6 | 23.7 | 13.7 | 1.5 | 0.1 | 2.4 | 4.9 | 3.6 | 0.0 | 0.61 |
| **246 bulk** | 4.2 | 3.3 | 5.7 | 8.1 | 5.1 | 5.5 | 11.8 | 4.9 | 14.2 | 37.1 | 0.0 | 6.7 |
| **246-3** | 3.7 | 2.3 | 6.4 | 9.8 | 4.9 | 4.2 | 10.7 | 3.3 | 14.1 | 40.7 | 0.0 | 12.6 |
| **246-2** | 5.5 | 6.5 | 6.0 | 9.5 | 5.3 | 5.0 | 13.5 | 5.6 | 13.1 | 30.0 | 0.0 | 4.7 |
| **22-4** | 8.3 | 7.6 | 8.3 | 10.3 | 5.3 | 11.1 | 7.0 | 4.7 | 13.7 | 20.4 | 3.3 | 0.4 |
| **22-3** | 7.9 | 9.5 | 3.1 | 7.9 | 3.5 | 12.6 | 1.3 | 13.8 | 14.4 | 25.9 | 0.0 | 0.5 |
| **8-3** | 5.5 | 5.5 | 10.0 | 13.8 | 8.9 | 2.4 | 15.9 | 5.4 | 10.6 | 22.0 | 0.0 | 0.9 |
| **97-7** | 9.1 | 24.7 | 9.3 | 16.7 | 8.9 | 3.8 | 5.4 | 4.6 | 6.8 | 10.7 | 0.0 | 0.4 |
| **97-6** | 11.6 | 18.2 | 11.1 | 16.8 | 9.1 | 3.5 | 6.4 | 4.0 | 5.6 | 13.7 | 0.0 | 0.5 |
| **97-5** | 5.7 | 7.9 | 11.1 | 16.9 | 8.4 | 4.8 | 9.7 | 6.8 | 11.6 | 17.2 | 0.0 | 1.0 |
| **97-3-4** | 4.7 | 5.4 | 9.5 | 13.6 | 7.2 | 8.5 | 2.2 | 7.1 | 15.3 | 26.4 | 0.0 | 2.3 |
| **97-2** | 4.4 | 4.9 | 8.9 | 12.1 | 6.4 | 10.9 | 7.9 | 6.3 | 13.4 | 24.8 | 0.0 | 4.3 |
| **108-4** | 12.4 | 15.7 | 13.4 | 14.9 | 9.4 | 5.3 | 5.2 | 5.4 | 10.3 | 8.0 | 0.0 | 0.32 |
| **108-3** | 14.9 | 16.5 | 14.6 | 21.0 | 11.6 | 4.2 | 1.1 | 1.6 | 3.4 | 11.2 | 0.0 | 0.18 |
| **2400-bulk** | 5.5 | 7.0 | 6.1 | 8.4 | 5.0 | 5.4 | 4.8 | 8.5 | 21.5 | 27.5 | 0.4 | 10.2 |
| **2400-4** | 5.4 | 9.8 | 5.5 | 5.4 | 3.5 | 5.5 | 10.1 | 5.7 | 21.8 | 26.6 | 0.7 | 2.6 |
| **2400-3** | 6.4 | 6.7 | 7.2 | 11.8 | 12.0 | 7.2 | 8.3 | 3.5 | 13.0 | 23.3 | 0.5 | 9.6 |
| **2400-1** | 14.1 | 6.3 | 6.1 | 9.7 | 4.2 | 4.1 | 10.6 | 1.3 | 15.2 | 27.9 | 0.5 | 2.0 |
| **172a-bulk** | 11.7 | 7.8 | 5.3 | 6.5 | 3.8 | 11.7 | 11.5 | 6.0 | 12.4 | 23.3 | 0.0 | 2.0 |
| **172a-2** | 5.3 | 5.7 | 7.9 | 11.0 | 6.7 | 9.9 | 8.9 | 4.7 | 12.7 | 27.4 | 0.0 | 2.5 |
| **172a-3** | 11.3 | 7.7 | 6.3 | 10.1 | 4.6 | 10.9 | 8.0 | 2.9 | 10.2 | 27.5 | 0.5 | 0.9 |
| **172-4** | 8.6 | 19.6 | 8.3 | 15.3 | 8.3 | 2.9 | 7.3 | 6.2 | 9.2 | 14.3 | 0.0 | 0.2 |
| **96-6** | 6.5 | 8.2 | 9.7 | 14.5 | 7.7 | 7.7 | 14.5 | 7.9 | 7.0 | 16.0 | 0.0 | 1.3 |
| **168-3** | 13.0 | 14.5 | 10.3 | 15.9 | 9.0 | 5.5 | 4.0 | 3.9 | 6.8 | 17.0 | 0.0 | 0.1 |
| **168-2** | 8.5 | 10.3 | 12.1 | 19.6 | 9.4 | 5.8 | 6.2 | 6.9 | 9.3 | 11.9 | 0.0 | 0.4 |
