# Supplementary material for: GC/MS and proteomics to unravel the painting history of the lost Giant Buddhas of Bāmiyān (Afghanistan)
Source: PLoS One. 2017 Apr 5;12(4):e0172990. doi: 10.1371/journal.pone.0172990 (PMC5381772; doi:10.1371/journal.pone.0172990)
Supplement: S5 File — (DOCX) [file pone.0172990.s005.docx]

**GC/MS and Proteomics to unravel the painting history of the lost Giant Buddhas of Bāmiyān (Afghanistan)**

Anna Lluveras-Tenorio, Roberto Vinciguerra, Eugenio Galano, Catharina Blaensdorf, Erwin Emmerling, Maria Perla Colombini, Leila Birolo, Ilaria Bonaduce

**S5 LC-MS/MS analyses**

Proteins were identified searching UniprotSprot database with MSMS Ion search Mascot software (Matrix Science) with Chordata as taxonomy restriction. with deamidation on Gln and Asn, oxidation on Met, pyro-Glu formation at Gln at the N-terminus of peptides as variable modifications, and a peptide mass tolerance of 10 ppm. Individual ions scores > 32 indicate identity or extensive homology (p<0.05). Only proteins identified with at least two peptides were considered as significative. In Table S5, peptide sequences are given with preceding and following residues. Underlined peptide sequences are unique to the specific species, while the others are in common between homologous proteins.

**Table S5. Identification of the proteins in the samples by LC-MSMS after trypsin digestion in heterogeneous phase**.

| SAMPLE 206-6-7-4 |  |  |  |
| --- | --- | --- | --- |
| Identified protein  (Accession number) | **Sequence coverage** (%) | **Individual Ion Score** | **Matched sequence** |
| Alpha-S2-casein  *Capra hircus* (P33049) | **13** | 20  34  13  17  20 | K.TNAIPYVR.Y + Deamidated (NQ)  R.NAGPFTPTVNR.E + Deamidated (NQ)  R.NAGPFTPTVNR.E + 2 Deamidated (NQ)  R.NANEEEYSIR.S  R.NANEEEYSIR.S + Deamidated (NQ) |

| SAMPLE 206-4-3 |  |  |  |
| --- | --- | --- | --- |
| Identified protein  (Accession number) | **Sequence coverage** (%) | **Individual Ion Score** | **Matched sequence** |
| Alpha-S1-casein  *Bos taurus* (P02662) | **21** | 27  23  13 | R.YLGYLEQLLR.L + Deamidated (NQ)  R.FFVAPFPEVFGK.E  K.HQGLPQEVLNENLLR.F + 3 Deamidated (NQ) |

| SAMPLE 188 |  |  |  |
| --- | --- | --- | --- |
| Identified protein  (Accession number) | **Sequence coverage** (%) | **Individual Ion Score** | **Matched sequence** |
| Alpha-S1-casein  *Bos taurus* (P02662)  or *Bubalis bubalis* (O62823) | **13** | 24  28  22  17 | K.EDVPSER.Y  R.YLGYLEQLLR.L  R.YLGYLEQLLR.L + Deamidated (NQ)  R. FFVAPFPEVFGK.E |
| Alpha-S1-casein  *Capra hircus* (P18626)  or *Ovis aries* ( P04653 ) | **13** | 24  28  22  12 | K.EDVPSER.Y  R.YLGYLEQLLR.L  R.YLGYLEQLLR.L + Deamidated (NQ)  R. FVVAPFPEVFR.K |

| SAMPLE 235-bulk |  |  |  |
| --- | --- | --- | --- |
| Identified protein  (Accession number) | **Sequence coverage** (%) | **Individual Ion Score** | **Matched sequence** |
| Alpha-S1-casein  *Bos taurus* (P02662) | **27** | 16  19  28  28  21  17  16  47 | K.EKVNELSK.D  R.YLGYLEQLLR.L  R.YLGYLEQLLR.L + Deamidated (NQ)  R. FFVAPFPEVFGK.E  K.VPQLEIVPNSAEER.L  K.VPQLEIVPNSAEER.L + Deamidated (NQ)  K.VPQLEIVPNSAEER.L + 2 Deamidated (NQ)  K.HQGLPQEVLNENLLR.F + Deamidated (NQ) |
| Alpha-S1-casein  *Capra hircus* (P18626)  or *Ovis aries* (P04653) | **9** | 19  28  19 | R.YLGYLEQLLR.L  R.YLGYLEQLLR.L + Deamidated (NQ)  R. FVVAPFPEVFR.K |
| Alpha-S2-casein  *Bos taurus* (P02663) | **26** | 25  31  19  24  16  11  16 | K.FALPQYLK.T  K.FALPQYLK.T + Deamidated (NQ)  R.NAVPITPTLNR.E  R.NAVPITPTLNR.E + Deamidated (NQ)  R.EQLSTSEENSK.K  K.ALNEINQFYQK.F + Deamidated (NQ)  K.TVDMESTEVFTK.K + Oxidation (M) |
| Beta-casein  *Bos taurus*(P02666)  or *Bubalis bubalis* (Q9TSI0) | **17** | 14  12  18  20  16  20  24 | K.EMPFPK.Y + Oxidation (M)  K.VLPVPQK.A  K.AVPYPQR.D  K.AVPYPQR.D + Deamidated (NQ)  R.DMPIQAFLLYQEPVLGPVR.G + Oxidation (M)  R.DMPIQAFLLYQEPVLGPVR.G +Oxidation(M); Deamidated (NQ)  R.DMPIQAFLLYQEPVLGPVR.G +Oxidation(M); 2 Deamidated (NQ) |
| Beta-lactoglobulin  *Bos taurus* (P02754)  or *Bubalis bubalis* (P02755) | **19** | 15  57  31 | K.IDALNENK.V + Deamidated (NQ)  K.VLVLDTDYK.K  R.TPEVDDEALEK.F |

| SAMPLE 235-5 |  |  |  |
| --- | --- | --- | --- |
| Identified protein  (Accession number) | **Sequence coverage** (%) | **Individual Ion Score** | **Matched sequence** |
| Alpha-S1-casein  *Bos taurus* (P02662)  or *Bubalis bubalis* (O62823) | **13** | 24  19  18  8 | K.EDVPSER.Y  R.YLGYLEQLLR.L  R.YLGYLEQLLR.L + Deamidated (NQ)  R.FFVAPFPEVFGK.E |
| Alpha-S1-casein  *Capra hircus* (P18626)  or *Ovis aries* (P04653) | **13** | 24  19  18  20 | K.EDVPSER.Y  R.YLGYLEQLLR.L  R.YLGYLEQLLR.L + Deamidated (NQ)  R.FVVAPFPEVFR.K |

| SAMPLE 235-4 |  |  |  |
| --- | --- | --- | --- |
| Identified protein  (Accession number) | **Sequence coverage** (%) | **Individual Ion Score** | **Matched sequence** |
| Alpha-S1-casein  *Bos taurus* (P02662)  or *Bubalis bubalis* (O62823) | **13** | 40  18  30  23 | K.EDVPSER.Y  R.YLGYLEQLLR.L  R.YLGYLEQLLR.L + Deamidated (NQ)  R.FFVAPFPEVFGK.E |
| Alpha-S1-casein  *Capra hircus* (P18626)  or *Ovis aries* (P04653) | **13** | 40  18  30  23 | K.EDVPSER.Y  R.YLGYLEQLLR.L  R.YLGYLEQLLR.L + Deamidated (NQ)  R.FVVAPFPEVFR.K |

| SAMPLE 235--3 |  |  |  |
| --- | --- | --- | --- |
| Identified protein  (Accession number) | **Sequence coverage** (%) | **Individual Ion Score** | **Matched sequence** |
| Alpha-S1-casein  *Bos taurus* (P02662)  or *Bubalis bubalis* (O62823) | **13** | 40  32  33  26 | K.EDVPSER.Y  R.YLGYLEQLLR.L  R.YLGYLEQLLR.L + Deamidated (NQ)  R. FFVAPFPEVFGK.E |
| Alpha-S1-casein  *Capra hircus* (P18626)  or *Ovis aries* (P04653) | **13** | 40  32  33  15 | K.EDVPSER.Y  R.YLGYLEQLLR.L  R.YLGYLEQLLR.L + Deamidated (NQ)  R. FVVAPFPEVFR.K |
| Alpha-S2-casein  *Bos taurus* (P02663) | **10** | 27  30  14 | R.NAVPITPTLNR.E  R.NAVPITPTLNR.E + Deamidated (NQ)  R.NAVPITPTLNR.E + 2 Deamidated (NQ) |
| Beta-casein  *Bos taurus*(P02666)  or *Bubalis bubalis* (Q9TSI0) | **5** | 16  10  14 | K.EMPFPK.Y + Oxidation (M)  K.AVPYPQR.D  K.AVPYPQR.D + Deamidated (NQ) |

| SAMPLE 235—1 |  |  |  |
| --- | --- | --- | --- |
| Identified protein  (Accession number) | **Sequence coverage** (%) | **Individual Ion Score** | **Matched sequence** |
| Alpha-S1-casein  *Bos taurus* (P02662)  or  *Bubalis bubalis* (O62823) | **13** | 17  31  39  17 | K.EDVPSER.Y  R.YLGYLEQLLR.L  R.YLGYLEQLLR.L + Deamidated (NQ)  R. FFVAPFPEVFGK.E |
| Alpha-S1-casein  *Capra hircus* (P18626)  or *Ovis aries* (P04653) | **13** | 17  31  39  11 | K.EDVPSER.Y  R.YLGYLEQLLR.L  R.YLGYLEQLLR.L + Deamidated (NQ)  R. FVVAPFPEVFR.K |

| SAMPLE 18-4 |  |  |  |
| --- | --- | --- | --- |
| Identified protein  (Accession number) | **Sequence coverage** (%) | **Individual Ion Score** | **Matched sequence** |
| Alpha-S1-casein  *Bos taurus* (P02662)  or *Bubalis bubalis* (O62823) | **8** | 42  17 | K.EDVPSER.Y  R.FFVAPFPEVFGK.E |
| Beta-casein  *Bos taurus*(P02666)  or *Bubalis bubalis* (P02755) | **6** | 25  18 | K.VLPVPQK.A  K.AVPYPQR.D |

| SAMPLE 18-1 |  |  |  |
| --- | --- | --- | --- |
| Identified protein  (Accession number) | **Sequence coverage** (%) | **Individual Ion Score** | **Matched sequence** |
| Alpha-S1-casein  *Bos taurus* (P02662)  or *Bubalis bubalis* (O62823) | **8** | 20  17 | K.EDVPSER.Y  R.FFVAPFPEVFGK.E |

| SAMPLE 22-4 |  |  |  |
| --- | --- | --- | --- |
| Identified protein  (Accession number) | **Sequence coverage** (%) | **Individual Ion Score** | **Matched sequence** |
| Alpha-S1-casein  *Bos taurus* (P02662)  or *Bubalis bubalis* (O62823) | **8** | 28  16 | K.EDVPSER.Y  R.FFVAPFPEVFGK.E |

| SAMPLE 16-4 |  |  |  |
| --- | --- | --- | --- |
| Identified protein  (Accession number) | **Sequence coverage** (%) | **Individual Ion Score** | **Matched sequence** |
| Alpha-S1-casein  *Bos taurus* (P02662)  or *Bubalis bubalis* (O62823) | **8** | 28  11 | K.EDVPSER.Y  R.FFVAPFPEVFGK.E |
| Alpha-S1-casein  *Capra hircus* (P18626)  or *Ovis aries* (P04653) | **13** | 28  18 | K.EDVPSER.Y  R. FVVAPFPEVFR.K |

| SAMPLE 16-3 |  |  |  |
| --- | --- | --- | --- |
| Identified protein  (Accession number) | **Sequence coverage** (%) | **Individual Ion Score** | **Matched sequence** |
| Alpha-S1-casein  *Bos taurus* (P02662) | **13** | 30  16  16 | K.EDVPSER.Y  R.YLGYLEQLLR.L  R.FFVAPFPEVFGK.E |
| Alpha-S1-casein  *Capra hircus* (P18626)  or *Ovis aries* (P04653) | **13** | 30  16  23 | K.EDVPSER.Y  R.YLGYLEQLLR.L  R.FVVAPFPEVFR.K |
| Beta-casein  *Bos taurus*(P02666) | **5** | 14  11  16 | K.VLPVPQK.A  K.AVPYPQR.D  K.AVPYPQR.D + Deamidation (NQ) |

| SAMPLE 214-7 |  |  |  |
| --- | --- | --- | --- |
| Identified protein  (Accession number) | **Sequence coverage** (%) | **Individual Ion Score** | **Matched sequence** |
| Alpha-S1-casein  *Bos taurus* (P02662)  or *Bubalis bubalis* (O62823) | **13** | 30  34  10 | R.YLGYLEQLLR.L  R.YLGYLEQLLR.L + Deamidated (NQ)  R. FFVAPFPEVFGK.E |
| Alpha-S1-casein  *Capra hircus* (P18626)  or *Ovis aries* (P04653) | **13** | 30  34  20 | R.YLGYLEQLLR.L  R.YLGYLEQLLR.L + Deamidated (NQ)  R. FVVAPFPEVFR.K |
| Alpha-S2-casein  *Bos taurus* (P02663) | **8** | 11  24  13  15 | K.FALPQYLK.T + Deamidated (NQ)  R.NAVPITPTLNR.E  R.NAVPITPTLNR.E + Deamidated (NQ)  R.NAVPITPTLNR.E + 2 Deamidated (NQ) |
| Beta-casein  *Bos taurus*(P02666)  or *Bubalis bubalis* (Q9TSI0) | **5** | 19  17  16 | K.EMPFPK.Y + Oxidation (M)  K.AVPYPQR.D  K.AVPYPQR.D + Deamidated (NQ) |
| Beta-lactoglobulin  *Bos taurus* (P02754)  or *Bubalis bubalis* (P02755)  or *Ovis aries* (P67976) | **11** | 32  15 | K.VLVLDTDYK.K  R.TPEVDDEALEK.F |

| SAMPLE 214-2 |  |  |  |
| --- | --- | --- | --- |
| Identified protein  (Accession number) | **Sequence coverage** (%) | **Individual Ion Score** | **Matched sequence** |
| Alpha-S1-casein  *Bos taurus* (P02662) | **20** | 44  18  13  29  20  18 | K.EDVPSER.Y  R.YLGYLEQLLR.L  R.YLGYLEQLLR.L + Deamidated (NQ)  R.FFVAPFPEVFGK.E  K.VPQLEIVPNSAEER.L + Deamidated (NQ)  K.VPQLEIVPNSAEER.L + 2 Deamidated (NQ) |
| Alpha-S1-casein  *Capra hircus* (P18626)  or *Ovis aries* (P04653) | **13** | 44  18  13  35 | K.EDVPSER.Y  R.YLGYLEQLLR.L  R.YLGYLEQLLR.L + Deamidated (NQ)  R.FVVAPFPEVFR.K |
| Alpha-S2-casein  *Bos taurus* (P02663) | **21** | 10  22  16  27  20  20  17  12  15 | K.VIPYVR.Y  K.FALPQYLK.T  K.FALPQYLK.T + Deamidated (NQ)  R.NAVPITPTLNR.E  R.NAVPITPTLNR.E + Deamidated (NQ)  R.NAVPITPTLNR.E + 2 Deamidated (NQ)  K.ALNEINQFYQK.F + Deamidated (NQ)  K.ALNEINQFYQK.F + 2 Deamidated (NQ)  K.TVDMESTEVFTK.K + Oxidation (M) |
| Alpha-S2-casein  *Capra hircus* ( P33049) | **14** | 29  12  18  17  12 | R.NAGPFTPTVNR.E  R.NAGPFTPTVNR.E + 2 Deamidated (NQ)  R.NANEEEYSIR.S  K.ALNEINQFYQK.F + Deamidated (NQ)  K.ALNEINQFYQK.F + 2 Deamidated (NQ) |
| Beta-casein  *Bos taurus*(P02666) or  *Bubalus bubalis* (Q9TSI0) | **17** | 27  17  14 | K.VLPVPQK.A  K.AVPYPQR.D  R.DMPIQAFLLYQEPVLGPVR.G + 2Deamidated (NQ) |
| Beta-lactoglobulin  *Bos taurus* (P02754) or  *Bubalus bubalis* (P02755) | **15** | 19  24  61 | K.IDALNENK.V + Deamidated (NQ)  K.VLVLDTDYK.K  R.TPEVDDEALEK.F |

| SAMPLE 214-4 |  |  |  |
| --- | --- | --- | --- |
| Identified protein  (Accession number) | **Sequence coverage** (%) | **Individual Ion Score** | **Matched sequence** |
| Alpha-S1-casein  *Bos taurus* (P02662) | **20** | 27  30  38  18  16  26 | K.EDVPSER.Y  R.YLGYLEQLLR.L  R.YLGYLEQLLR.L + Deamidated (NQ)  R.FFVAPFPEVFGK.E  K.HQGLPQEVLNENLLR.F  K.HQGLPQEVLNENLLR.F + Deamidated (NQ) |
| Alpha-S1-casein  *Capra hircus* (P18626)  or *Ovis aries* (P04653) | **13** | 27  30  38  17 | K.EDVPSER.Y  R.YLGYLEQLLR.L  R.YLGYLEQLLR.L + Deamidated (NQ)  R. FVVAPFPEVFR.K |
| Alpha-S2-casein  *Bos taurus* (P02663) | **13** | 14  21  12 | K.FALPQYLK.T + Deamidated (NQ)  R.NAVPITPTLNR.E  R.NAVPITPTLNR.E + Deamidated (NQ) |
| Beta-casein  *Bubalis bubalis* (Q9TSI0) | **30** | 13  20  14  22 | K.VLPVPQK.A  K.AVPYPQR.D  K.AVPYPQR.D + Deamidated (NQ)  R.DMPIQAFLLYQEPVLGPVR.G+2 Deamidated (NQ); Oxidation (M) |
| Beta-lactoglobulin  *Bos taurus* (P02754)  or *Bubalis bubalis* (P02755) | **15** | 32  39 | K.VLVLDTDYK. K  R.TPEVDDEALEK.F |

Figures A and B show the amminoacidic sequences identified in the samples. The experimentally identified peptides are highlighted in grey. Strikethrough sequence is the signal peptide that is absent in the mature form of the protein. Protein sequences were aligned using Align tool available online at UniProt The UniProt Consortium, UniProt: a hub for protein information. Nucleic Acids Res. 43: D204-D212 (2015)), which uses Clustal-Omega program (Sievers F, Wilm A, Dineen DG, Gibson TJ, Karplus K, Li W, Lopez R, McWilliam H, Remmert M, Söding J, Thompson JD, Higgins DG (2011). Fast, scalable generation of high-quality protein multiple sequence alignments using Clustal Omega. Molecular Systems Biology 7:539 doi:10.1038/msb.2011.75).

1    ~~MKLLILTCLVAVALA~~RPKHPIKHQGLPQEVLNENLLRFFVAPFPEVFGKEKVNELSKDIG   60  [P02662](http://www.uniprot.org/uniprot/P02662)   CASA1_BOVIN

1    ~~MKLLILTCLVAVALA~~RPKQPIKHQGLPQGVLNENLLRFFVAPFPEVFGKEKVNELSTDIG   60  [O62823](http://www.uniprot.org/uniprot/O62823)   CASA1_BUBBU

1    ~~MKLLILTCLVAVALA~~RPKHPINHRGLSPEVPNENLLRFVVAPFPEVFRKENINELSKDIG   60  [P18626](http://www.uniprot.org/uniprot/P18626)   CASA1_CAPHI

1    ~~MKLLILTCLVAVALA~~RPKHPIKHQGLSSEVLNENLLRFVVAPFPEVFRKENINELSKDIG   60  [P04653](http://www.uniprot.org/uniprot/P04653)   CASA1_SHEEP

******************:**:*:**   * *******.******** **::****.***

61   SESTEDQAMEDIKQMEAESISSSEEIVPNSVEQKHIQKEDVPSERYLGYLEQLLRLKKYK  120  [P02662](http://www.uniprot.org/uniprot/P02662)   CASA1_BOVIN

61   SESTEDQAMEDIKQMEAESISSSEEIVPISVEQKHIQKEDVPSERYLGYLEQLLRLKKYN  120  [O62823](http://www.uniprot.org/uniprot/O62823)   CASA1_BUBBU

61   SESTEDQAMEDAKQMKAGSSSSSEEIVPNSAEQKYIQKEDVPSERYLGYLEQLLRLKKYN  120  [P18626](http://www.uniprot.org/uniprot/P18626)   CASA1_CAPHI

61   SESIEDQAMEDAKQMKAGSSSSSEEIVPNSAEQKYIQKEDVPSERYLGYLEQLLRLKKYN  120  [P04653](http://www.uniprot.org/uniprot/P04653)   CASA1_SHEEP

     *** ******* ***:* * ******** *.***:************************:

121  VPQLEIVPNSAEERLHSMKEGIHAQQKEPMIGVNQELAYFYPELFRQFYQLDAYPSGAWY  180  [P02662](http://www.uniprot.org/uniprot/P02662)   CASA1_BOVIN

121  VPQLEIVPNLAEEQLHSMKEGIHAQQKEPMIGVNQELAYFYPQLFRQFYQLDAYPSGAWY  180  [O62823](http://www.uniprot.org/uniprot/O62823)   CASA1_BUBBU

121  VPQLEIVPKSAEEQLHSMKEGNPAHQKQPMIAVNQELAYFYPQLFRQFYQLDAYPSGAWY  180  [P18626](http://www.uniprot.org/uniprot/P18626)   CASA1_CAPHI

121  VPQLEIVPKSAEEQLHSMKEGNPAHQKQPMIAVNQELAYFYPQLFRQFYQLDAYPSGAWY  180  [P04653](http://www.uniprot.org/uniprot/P04653)   CASA1_SHEEP

     ********: ***:*******  *:**:***.**********:*****************

181  YVPLGTQYTDAPSFSDIPNPIGSENSEKTTMPLW  214  [P02662](http://www.uniprot.org/uniprot/P02662)   CASA1_BOVIN

181  YVPLGTQYPDAPSFSDIPNPIGSENSGKTTMPLW  214  [O62823](http://www.uniprot.org/uniprot/O62823)   CASA1_BUBBU

181  YLPLGTQYTDAPSFSDIPNPIGSENSGKTTMPLW  214  [P18626](http://www.uniprot.org/uniprot/P18626)   CASA1_CAPHI

181  YLPLGTQYTDAPSFSDIPNPIGSENSGKITMPLW  214  [P04653](http://www.uniprot.org/uniprot/P04653)   CASA1_SHEEP

     *:****** ***************** * *****

**Figure A. Alignment of the sequences of Alpha S1 caseins.** The aminoacidic sequences from *Bos taurus* (P02662), *Bubalus bubalis* (O62823), *Capra hircus* (P18626) and *Ovis aries* (P04653). * (asterisk) indicates positions which have a single, fully conserved residue; : (colon) indicates conservation between groups of strongly similar properties - scoring > 0.5 in the Gonnet PAM 250 matrix; . (period) indicates conservation between groups of weakly similar properties - scoring =< 0.5 in the Gonnet PAM 250 matrix.

1    ~~MKFFIFTCLLAVALA~~KNTMEHVSSSEESI-ISQETYKQEKNMAINPSKENLCSTFCKEVV   59  [P02663](http://www.uniprot.org/uniprot/P02663)   CASA2_BOVIN

1    ~~MKFFIFTCLLAVALA~~KHKMEHVSSSEEPINIFQEIYKQEKNMAIHPRKEKLCTTSCEEVV   60  [P33049](http://www.uniprot.org/uniprot/P33049)   CASA2_CAPHI

     ****************..********* * * ** *********.* **:**:* *:***

60   RNANEEEYSIGSSSEESAEVATEEVKITVDDKHYQKALNEINQFYQKFPQYLQYLYQGPI  119  [P02663](http://www.uniprot.org/uniprot/P02663)   CASA2_BOVIN

61   RNANEEEYSIRSSSEESAEVAPEEIKITVDDKHYQKALNEINQFYQKFPQYLQYPYQGPI  120  [P33049](http://www.uniprot.org/uniprot/P33049)   CASA2_CAPHI

     ********** ********** **:***************************** *****

120  VLNPWDQVKRNAVPITPTLNREQLSTSEENSKKTVDMESTEVFTKKTKLTEEEKNRLNFL  179  [P02663](http://www.uniprot.org/uniprot/P02663)   CASA2_BOVIN

121  VLNPWDQVKRNAGPFTPTVNREQLSTSEENSKKTIDMESTEVFTKKTKLTEEEKNRLNFL  180  [P33049](http://www.uniprot.org/uniprot/P33049)   CASA2_CAPHI

     ************ *:***:***************:*************************

180  KKISQRYQKFALPQYLKTVYQHQKAMKPWIQPKTKVIPYVRYL  222  [P02663](http://www.uniprot.org/uniprot/P02663)   CASA2_BOVIN

181  KKISQYYQKFAWPQYLKTVDQHQKAMKPWTQPKTNAIPYVRYL  223  [P33049](http://www.uniprot.org/uniprot/P33049)   CASA2_CAPHI

     ***** ***** ******* ********* ****:.*******

**Figure B. Alignment of the sequences of Alpha S2 caseins.** The aminoacidic sequences from *Bos taurus* (P02663), *Capra hircus* (P33049). * (asterisk) indicates positions which have a single, fully conserved residue; : (colon) indicates conservation between groups of strongly similar properties - scoring > 0.5 in the Gonnet PAM 250 matrix; . (period) indicates conservation between groups of weakly similar properties - scoring =< 0.5 in the Gonnet PAM 250 matrix.
